# Grouping groupers in the Mediterranean: Ecological baselines revealed by ancient proteins

**DOI:** 10.1002/ece3.10625

**Published:** 2023-10-23

**Authors:** Rachel M. Winter, Willemien de Kock, Meaghan Mackie, Max Ramsøe, Elena Desiderà, Matthew Collins, Paolo Guidetti, Samantha Presslee, Marta Munoz Alegre, Tarek Oueslati, Arturo Morales Muniz, Dimitris Michailidis, Youri van den Hurk, Alberto J. Taurozzi, Canan Çakirlar

**Affiliations:** ^1^ Groningen Institute of Archaeology University of Groningen Groningen The Netherlands; ^2^ Marine Evolution and Conservation, Groningen Institute for Evolutionary Life Sciences University of Groningen Groningen The Netherlands; ^3^ Faculty of Health and Medical Science, The Globe Institute University of Copenhagen Copenhagen Denmark; ^4^ Faculty of Health and Medical Science, Novo Nordisk Foundation Center for Protein Research University of Copenhagen Copenhagen Denmark; ^5^ Department of Integrative Marine Ecology (EMI) Stazione Zoologica Anton Dohrn–National Institute of Marine Biology, Ecology and Biotechnology—Genoa Marine Centre Genoa Italy; ^6^ Department of Archaeology University of Cambridge Cambridge UK; ^7^ BioArCh, Department of Archaeology University of York York UK; ^8^ Centre National de la Recherche Scientifique University of Lille Lille France; ^9^ Department of Biology Universidad Autónoma de Madrid Madrid Spain; ^10^ Malcolm H. Wiener Lab, American School of Classical Studies at Athens Athens Greece; ^11^ Department of Archaeology and Cultural History Norwegian University of Science and Technology Trondheim Norway

**Keywords:** fisheries, groupers (Epinephelidae), marine ecology, marine historical ecology, Mediterranean, paleoproteomics, zooarchaeology

## Abstract

Marine historical ecology provides a means to establish baselines to inform current fisheries management. Groupers (Epinephelidae) are key species for fisheries in the Mediterranean, which have been heavily overfished. Species abundance and distribution prior to the 20th century in the Mediterranean remains poorly known. To reconstruct the past biogeography of Mediterranean groupers, we investigated whether Zooarchaeology by Mass Spectrometry (ZooMS) can be used for identifying intra‐genus grouper bones to species level. We discovered 22 novel, species‐specific ZooMS biomarkers for groupers. Applying these biomarkers to Kinet Höyük, a Mediterranean archaeological site, demonstrated 4000 years of regional *Epinephelus aeneus* dominance and resiliency through millennia of fishing pressures, habitat degradation and climatic changes. Combining ZooMS identifications with catch size reconstructions revealed the *Epinephelus aeneus* capacity for growing 30 cm larger than hitherto documented, revising the maximum Total Length from 120 to 150 cm. Our results provide ecological baselines for a key Mediterranean fishery which could be leveraged to define and assess conservation targets.

## Graphical abstract

Groupers (Epinephelidae) are key species for fisheries in the Mediterranean which have been heavily overfished. Species abundance and distribution prior to the 20th century in the Mediterranean remains poorly known. By using Zooarchaeology by Mass Spectrometry (ZooMS) and reconstructing collagen sequences, we discovered and validated 22 biomarkers for species‐level identifications of Mediterranean groupers. Applying these biomarkers to an archaeological case study revealed the millennia long resilience of the white grouper in the eastern Mediterranean and revised the growth capacity for this species by +25% (from 120 cm Total Length to 150 cm).

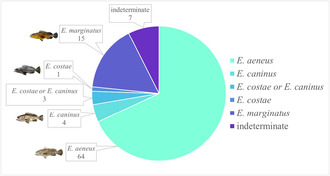

## 1 INTRODUCTION

Marine resources have been exploited for millennia, requiring extremely long‐term historical perspectives to understand how our current ecosystems have shifted compared to the baselines of the past (Pauly, 1995). Groupers are large, predatory fishes of high ecological and commercial importance worldwide. In the Mediterranean, we have been fishing groupers (Epinephelidae) for over 10,000 years and they have been heavily overfished in recent decades (Sadovy de Mitcheson et al., 2013; Sala, 2004). Zooarchaeological evidence testifies to the large sizes (>100 cm Total Length [TL]) that groupers reached in the past 5000 years and localised overfishing by the Hellenistic period (Winter et al., 2022). Whether overfishing or other anthropogenic impacts have caused changes in species abundance, maximum body size and geographical ranges of Mediterranean groupers remains unknown. Due to similar osteomorphology, it is exceptionally rare to be able to identify archaeological grouper bones to a species level (Desse & Desse‐Berset, 1996). In this study, we set out to investigate if Zooarchaeology by Mass Spectrometry (ZooMS; Buckley et al., 2009) is a viable method for species‐level identifications of Mediterranean groupers within the *Epinephelus* genus.

ZooMS uses peptide mass fingerprinting of collagen type I (hereafter COL1) for taxonomic identification (Buckley et al., 2009). Tryptic digestion of collagen cleaves the COL1 protein down into peptides. The variation in COL1 amino acid sequences between fauna results in peptides of different masses. For ZooMS, COL1 is analysed via matrix‐assisted laser desorption/ionisation time‐of‐flight (MALDI‐TOF) mass spectrometry, generating spectra with peaks which correspond to ZooMS biomarkers with different peptide masses. New ZooMS biomarkers are validated using liquid chromatography–tandem mass spectrometry (LC–MS/MS) to ensure they derive from COL1 and not another protein or contaminant. Whereas ZooMS by MALDI‐TOF analysis measures whole peptide masses after tryptic digestion, LC–MS/MS also fragments the peptides and measures the resulting fragment ions. With this additional step, the mass of each individual amino acid can be determined, enabling the reconstruction of protein amino acid sequences. Applying ZooMS to identify archaeological fish bones is severely limited by the availability of collagen sequences of many fish species (Warinner et al., 2022) as demonstrated by genus level ZooMS identifications of Caribbean grouper bones from archaeological contexts (Harvey et al., 2022). The absence of available reference data for our study species therefore required reconstructing COL1 sequences for our four grouper species using LC–MS/MS protein sequencing.

**FIGURE 1:**
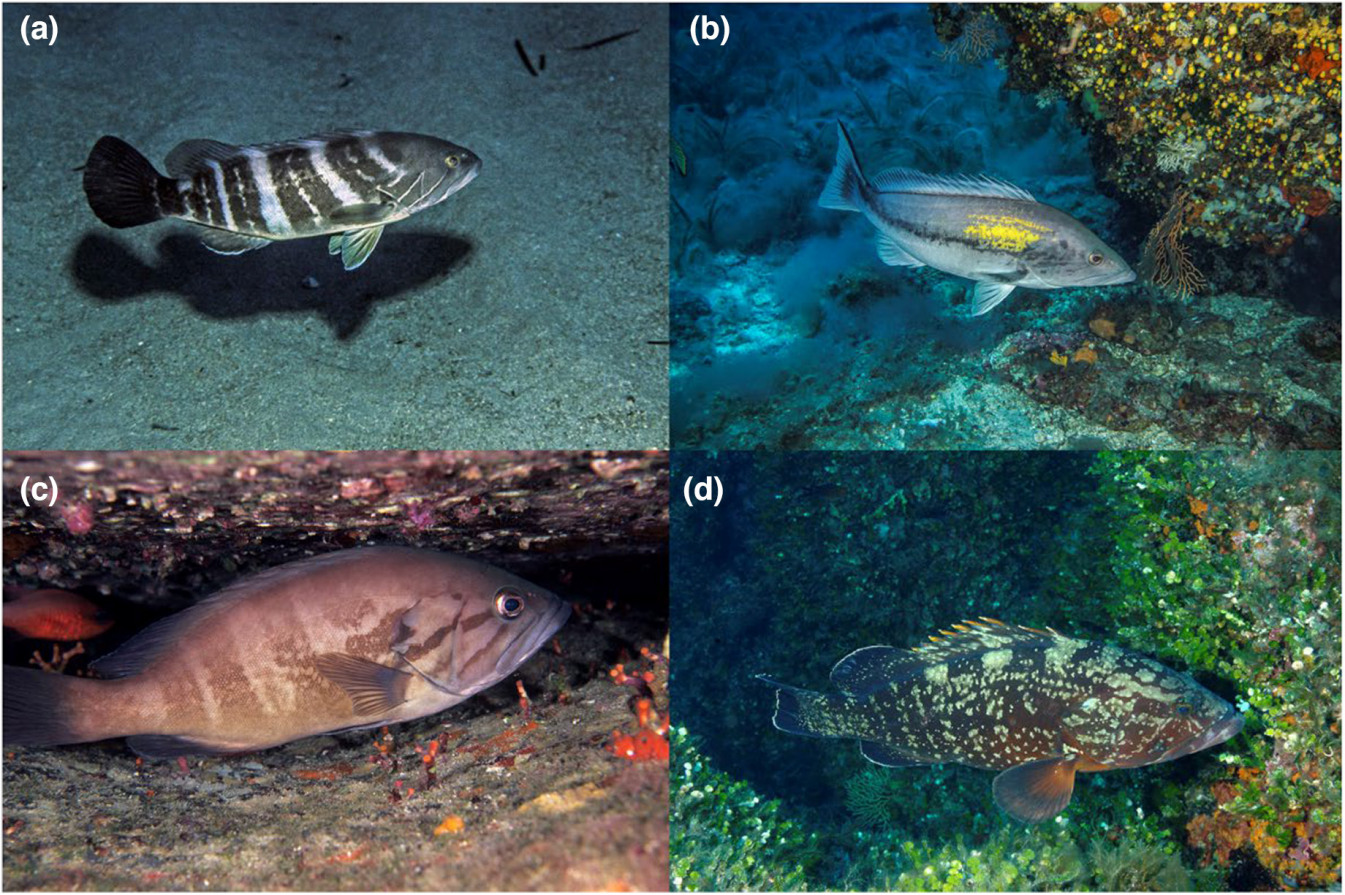
Four species of Mediterranean groupers within the *Epinephelus* genus: (a) white grouper (*E. aeneus*), (b) goldblotch grouper (*E. costae*), (c) dogtooth grouper (*E. caninus*) and (d) dusky grouper (*E. marginatus*). All photographs have been provided by and are © Egidio Trainito.

In fact, there are six species of groupers indigenous to the Mediterranean Sea with four of them belonging to the *Epinephelus* genus (Craig et al., 2011; Figure 1). As upper trophic level fishes in Mediterranean rocky reef ecosystems, groupers are ecologically important for maintaining balanced and healthy food webs (Prato et al., 2013). Mediterranean groupers have different ecological niches with interspecific differences regarding habitats, trophic resources and spawning patterns (Aronov & Goren, 2008; Desidera et al., 2019; Desiderà et al., 2021; Gökçe et al., 2003). *Epinephelus marginatus* (dusky grouper) is generally beholden as the most abundant grouper species in the Mediterranean and is thus the most studied species (Condini et al., 2018). Mediterranean groupers exhibit high site fidelity and small home ranges ca. 3–5 km (Desiderà et al., 2021; Di Franco et al., 2018; Fennessy, 2006; Hackradt et al., 2014; Sadovy de Mitcheson et al., 2013). These factors combined with interspecific ecological niches lead to regional variations in grouper species richness and abundance throughout the Mediterranean basin (Aronov & Goren, 2008; Desiderà et al., 2022; Glamuzina et al., 2002).

Groupers have been one of the most important taxa to eastern Mediterranean fisheries since at least the Neolithic period (Desse & Desse‐Berset, 1994, 1999) and remain among the most important target species to Turkish fisheries today (Mavruk et al., 2020; Ünal et al., 2009). The highest grouper landings based on caught biomass in Turkey come from Iskenderun Bay and the Gulf of Mersin (Mavruk, 2020). In present‐day Iskenderun Bay, local fishers report *Epinephelus aeneus* and *Epinephelus marginatus* are the most abundant grouper species (Mavruk et al., 2018), with *Epinephelus aeneus* composing the highest biomass from comber (Serranidae) and grouper catches (Özyurt & Kiyağa, 2016; Yemisken et al., 2014). The IUCN lists *Epinephelus aeneus* as Near Threatened throughout its global distribution and *Epinephelus marginatus* as Vulnerable in the Mediterranean basin (Pollard et al., 2018). Mediterranean groupers are highly territorial fishes and often display spatial and temporal differences in spawning activities (Aronov & Goren, 2008; Desidera et al., 2019; Desiderà et al., 2021; Gökçe et al., 2003), making the likelihood of wild hybridisation between them low.

During underwater visual census assessments and/or when assessing fisheries' catches, different species can be distinguished from each other (Guidetti et al., 2014; Mavruk et al., 2018). However, when presented with their bones, especially from archaeological contexts which regularly produce fragmentary remains, species‐level identifications are exceptionally rare (Desse & Desse‐Berset, 1996). When working with ichthyoarchaeological assemblages in the Mediterranean, in addition to groupers there are three species of combers (Serranidae) which must also be considered due to indistinguishable osteomorphology (Desse & Desse‐Berset, 1996), despite belonging to different taxonomic families (Craig et al., 2011; Vella et al., 2022; Vella & Vella, 2016). Combers have similar ecology to groupers and overlap in size with sexually immature groupers (Froese & Pauly, 2023).

Knowledge of present‐day species distribution and abundance is limited to well‐studied regions, with much of the work done within the high concentration of Marine Protected Areas (MPAs) of the northwestern Mediterranean (Andrello et al., 2013; Sala et al., 2012) and the catches of Mediterranean fisheries (Colloca et al., 2017; Condini et al., 2018; Pinnegar et al., 2003). Insight regarding grouper species' past geographical ranges is currently only informed by interpretations of historical texts and ancient artwork (Guidetti & Micheli, 2011; Pinnegar & Engelhard, 2008). Past temporal and geographical abundances and distributions of Mediterranean groupers would enable the reconstruction of historical ecological baselines, informing how interspecies competition and local ecosystems may have changed over time. Where historical baselines are lacking, as they are for much of the Mediterranean Sea, long established and well‐enforced MPAs are used for comparison with unprotected areas to assess marine conservation measures' effectiveness (Sala et al., 2012). Ichthyoarchaeolgical remains are the most tangible line of evidence for capturing the characteristics of these ecosystems in more ‘pristine’ forms.

Here we reconstruct four COL1 sequences and present 22 novel ZooMS biomarkers for intra‐genus species‐level identification of Mediterranean groupers. By applying these ZooMS biomarkers to an archaeological case study of Kinet Höyük, Turkey, we show millenia of *Epinephelus aeneus* dominance in northeastern Mediterranean archaeological assemblages and upwardly revise current knowledge of the maximum body size of *Epinephelus aeneus*.

## 2 MATERIALS AND METHODS

### 2.1 Archaeological fish remains and modern reference material

Archaeological grouper samples (*n* = 94) for ZooMS analysis come from occupational phases of Kinet Höyük spanning ca. 2400 BC–1400 AD (Table S1). Kinet Höyük is a coastal settlement site located in present‐day Turkey in the northeastern corner of the Mediterranean in Iskenderun Bay (36.8536° N, 36.1572° E; Çakırlar et al., 2021; Gates, 2013, 2015). Archaeological, often fragmented, fish bones were visually identified as groupers or combers using the modern reference collection at the Groningen Institute of Archaeology. We estimated catch sizes where possible using osteometrics, see Winter et al. (2022) for methodological details.

Modern reference specimens came from several zooarchaeological reference collections in the Mediterranean and encompassed the four species of Mediterranean groupers from the *Epinephelus* genus. Most of the reference specimens, 13 of 15, when collected (whole from fish markets) were identified by experienced ichthyologists (T.O. and A.M.M.) and the final two were identified by a non‐specialist using Whitehead et al. (1986). Acknowledging the possibility of false identifications, we used a minimum of three samples for each species obtained from at least two different collections. We were unable to obtain reference samples for the remaining two native Mediterranean grouper species (*Mycteroperca rubra* and *Hyporthodus haifensis*) and thus focused on testing the taxonomic intra‐genus resolution possible for ZooMS. We had bone samples from five *Epinephelus marginatus*, three *Epinephelus aeneus*, three *Epinephelus caninus* and three *Epinephelus costae* (Table S2). In addition to obtaining modern reference specimens for novel peptide marker detection and validation, genomic data of our study species have not been published. Manual reconstruction of the COL1 amino acid sequences for the four *Epinephelus* spp. in our study was thus necessary and alludes to the challenges of inadequate reference data available for the furthering of ZooMS work on fishes.

### 2.2 Methods

#### 2.2.1 Collagen extraction and data acquisition

Collagen extraction followed the protocol detailed in Buckley et al. (2009) with minor modification (see Data S1 for full method). ZooMS samples were analysed using a calibrated ultrafleXtreme (Bruker Daltonics) MALDI‐TOF instrument in reflector mode. For the LC–MS/MS data, all reference samples were analysed on a 77 min gradient with an EASY‐nLC 1200 (Proxeon) connected to an Exploris 480 Orbitrap mass spectrometer (Thermo Scientific). Additional details of LC–MS/MS data acquisition are provided in the Data S1.

#### 2.2.2 Reconstruction of COL1 amino acid sequences

The LC–MS/MS data, in the form of Thermo RAW files, from our Mediterranean *Epinephelus* spp. were first analysed against our reference library in pFind (version 3.1.6; Chi et al., 2018). Our reference library used in pFind was created using available fish collagen type I sequences found on UniProt and NCBI. Mediterranean grouper LC–MS/MS data were compared with the reference library to detect possible amino acid substitutions between our samples and the reference library. Searches in pFind were run with peptide mass tolerance ±10 ppm and fragment mass tolerance ±0.05 Da.

Hypothetical COL1 amino acid sequences were created for each of our four Mediterranean grouper species based off of proposed substitutions in pFind. Libraries containing our hypothetical COL1 sequences and unmodified fish COL1 sequences were searched against our LC–MS/MS data in MaxQuant (version 2.1.4.0, search parameters in Data S1) to verify amino acid coverage in our hypothetical sequences. Amino acids were only considered confidently identified if they occurred in two overlapping peptides.

#### 2.2.3 Identification and validation of ZooMS markers

MALDI‐TOF data for both the modern and archaeological samples were visualised in mMass (version 5.50). Baseline corrections and smoothing were applied with the default settings. Peaks were picked using a signal to noise ratio of 3.0–5.0 and then manually inspected. Where informative peptides were present, archaeological samples which did not meet this threshold were still considered. Masses of distinctive peaks in mMass found in modern samples were compared to the respective LC–MS/MS data in MaxQuant to confirm the mass and coverage of species‐specific peptide biomarkers. Discriminating peptide biomarkers were searched in NCBI BLASTp to ensure they were not the product of contamination in the laboratory and are not found in other Mediterranean fish with available collagen sequences.

#### 2.2.4 Constructing Mediterranean *Epinephelus* spp. phylogenetic relationships

Reconstructed Mediterranean grouper COL1 amino acid sequences were analysed in MEGA11 (Tamura et al., 2021) after aligning the sequences and determining which phylogenetic tree model best suited our data. The final tree was produced using a mtREV24 + G + F model and 10,000 replicates (bootstrapping). COL1 sequences of the gilthead seabream (*Sparus aurata*) and the European perch (*Perca fluviatilis*), both also within the Perciformes order, were used as the outgroup.

## 3 RESULTS

### 3.1 Phylogenetic tree for Mediterranean groupers from reconstructed COL1 sequences (mature chain coverage = 90%–95%)

Concatenated COL1 sequences were reconstructed for our four Mediterranean groupers and are provided in Data S1. These sequences provide useful reference data for future proteomic and ZooMS studies on groupers. We were able to reconstruct about 90%–95% of the mature portion of each protein chain (Table 1).

**TABLE 1:** Percentage of collagen type I sequences of Mediterranean *Epinephelus* spp. which were able to be reconstructed from modern LC–MS/MS data.

	COL1a1a coverage (%)		COL1a2 coverage (%)		COL1a1b coverage (%)	
	Total chain	Mature chain	Total chain	Mature chain	Total chain	Mature chain
*E. caninus*	75.47	96.76	81.05	94.99	71.56	91.15
*E. aeneus*	69.66	88.00	81.49	91.25	71.63	93.51
*E. marginatus*	74.22	96.76	83.04	96.26	69.35	91.15
*E. costae*	70.01	89.97	80.09	90.56	60.67	81.32

*Note*: Amino acids were only considered well covered if they were recovered in at least two unique, razor peptides. Amino acids which were only recovered in one peptide are thus not included in calculating the values above. The start of the mature peptide chain was determined following Brown et al. (2021).

**FIGURE 2:**
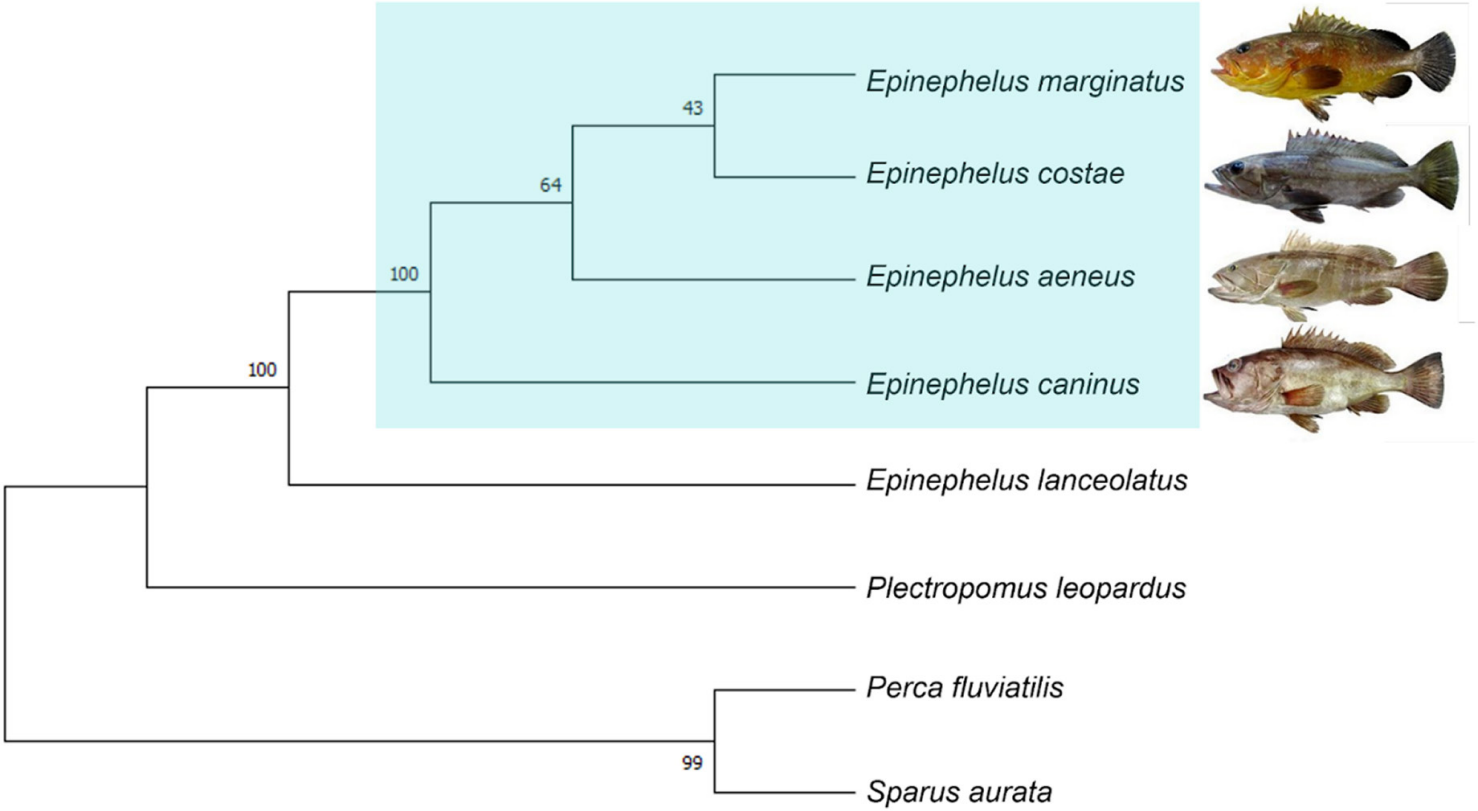
Phylogenetic tree of Mediterranean groupers in the *Epinephelus* genus produced using MEGA11 (Tamura et al., 2021), see Data S1 for full parameters of analysis. Mediterranean *Epinephelus* spp. images modified from Doğdu and Turan (2016). The highlighted turquoise box encompasses the four *Epinephelus* spp. indigenous to the Mediterranean Sea.

The phylogenetic tree supports our taxonomic identifications and indicates the four groupers are separated to species level, indicating adequate COL1 sequence variation between species for ZooMS to be a viable method for species ID. Our tree (Figure 2) is consistent with previous phylogenetic work on Epinephelidae (Vella et al., 2022; Vella & Vella, 2016, 2021). The bootstrap replicate values show high support for all but one of the proposed splits, with lower support for the taxonomic relation between *Epinephelus marginatus* and *Epinephelus costae*.

### 3.2 Validation of 22 ZooMS peptide biomarkers

**FIGURE 3:**
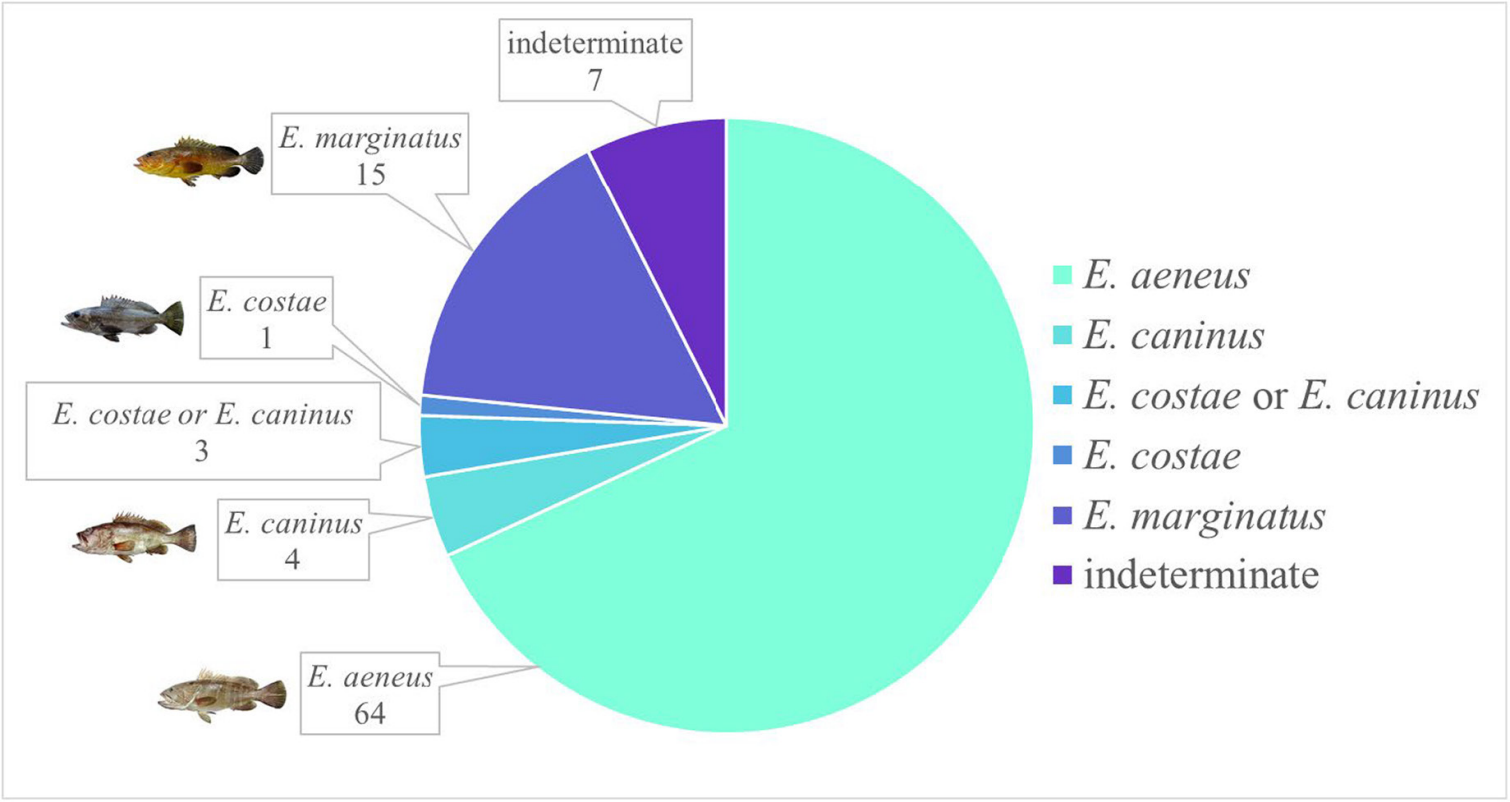
ZooMS identifications of Epinephelidae bones from Kinet Höyük (see Table S1). Mediterranean *Epinephelus* spp. images taken and modified from Doğdu and Turan (2016).

We discovered 22 novel COL1 ZooMS peptide biomarkers that can be used for species‐level identification of Mediterranean groupers within the *Epinephelus* genus (Table 2). Only biomarkers, which were not observed in all four study species, were present in the Thermo RAW files, and at least one form of the peptide could be visualised in the MALDI spectra have been included. At least one discriminating biomarker was found for all four species with three species‐specific biomarkers for *Epinephelus costae* and four for *Epinephelus caninus* (Figure 3). The spectra obtained from samples which were macerated with papain did not appear abnormal whereas the sample prepared with Neutrase did display a poor signal to noise ratio.

**TABLE 2:** Discriminating ZooMS biomarkers found in Mediterranean *Epinephelus*.

Mass (m/z)	Peptide sequence	Peptide name	*E. aeneus*	*E. costae*	*E. marginatus*	*E. caninus*
1319.6^a^	GE**G**GHRGPDGNAGR	COL1ɑ2 568				**x**
1350.6	GE**A**GHRGPDGNAGR	COL1ɑ2 568	x	x	x	x^b^
1755.78	GFTGMQGLPGPAG**A**HGER	COL1ɑ3 934	x		x	x
1783.8/1799.8	GFTGMQGLPGPAG**V**HGER	COL1ɑ3 934		**x**		
1965.9	GEPGPAGVQGL**S**GPSGEEGKR	COL1ɑ3 271	x^b^	x^b^	x	x^b^
1991.9	GEPGPAGVQGL**P**GPSGEEGKR	COL1ɑ3 271	x	x		x
2157.0	P**S**GPAGPAGQSGPPGASGPAGPTGAR	COL1ɑ2 662				**x**
2178.0	GF**S**GLPGPAGE**P**GKPGPSGPGGER	COL1ɑ1 793	x^b^		x	
2178.0	GF**P**GLPGPAGE**A**GKPGPSGPGGER	COL1ɑ1 793	x		x^b^	
2309.1	GLPGSPGSSGPPGKEG**A**AGP**A**GQDGR	COL1ɑ2 361	x^c^		x	x
2351.1	GLPGSPGSSGPPGKEG**P**AGP**S**GQDGR	COL1ɑ2 361		**x**		
2542.97	VGPPGPSGNPGPPGPAGG**T**GKEGPKGNR	COL1ɑ1 705			**x**	
2537.6	VGPPGPSGNPGPPGPAGG**P**GKEGPKGNR	COL1ɑ1 705	x^c^	x^c^		x^c^
2731.1	GFTGMQGLPGPAG**A**HGERGPAGASGPAGPR	COL1ɑ3 934	x		x	x
2775.0	GFTGMQGLPGPAG**V**HGERGPAGASGPAGPR	COL1ɑ3 934		**x**		
2851.34/2867.34	GLTGPLGLPGPAGATGDKGE**P**GPAGPVGP**G**GAR	COL1ɑ1 586		x	x	x
2855.33/2871.33	GLTGPLGLPGPAGATGDKGE**S**GPAGPVGP**A**GAR	COL1ɑ1 586	**x**			
2814.4	GPAGAQGAVGAPGPKGN**S**GDPGASGPKGEPGAK	COL1ɑ3 238		x^c^		
2889.2	GPAGAQGAVGAPGPKGN**N**GDPGASGPKGEPGAK	COL1ɑ3 238	x^c^		x	x
2915.3	GPPGPMGPPGLAGAPGEPGREGSPG**N**EGSAGR	COL1ɑ1 817	x^c^			
2947.3	GPPGPMGPPGLAGAPGEPGREGSPG**N**EGSAGR	COL1ɑ1 817	x^c^	x	x^b^	
2867.3	GPPGPMGPPGLAGAPGEPGREGSPG**N**EGSAGR	COL1ɑ1 817		x	x^b^	x
2888.3	GPPGPMGPPGLAGAPGEPGREGSPG**S**EGSAGR	COL1ɑ1 817			x^c^	

*Note*: Peptide names follow nomenclature proposed by Brown et al. (2021). Bolded values indicate peptide sequences and masses that are only found in one of our four intra‐genus grouper species.

^a^ This mass, but not necessarily this peptide sequence, is also found in a species of comber (*Serranus tigrinus*) in the Caribbean (see Harvey et al., 2022) and thus should be used in combination with other biomarkers for identifications.

^b^ Indicates a peak that is present in the MALDI due to the existence of an isobaric peptide.

^c^ Denotes a peptide which is present in the LC–MS/MS data but which is unable to be visualised in the MALDI‐TOF data.

Upon initial assessment and visualisation of MALDI‐TOF and LC–MS/MS data, it was apparent that one of the reference specimens (WLAp 1086) did not share many peaks with the other modern samples and was poorly covered by the *Epinephelus lanceolatus* collagen sequence in the reference library. Subsequent osteomorphological analysis of the individual this sample came from revealed that this fish was instead a seabass (*Dicentrarchus* sp.) as opposed to a grouper.

### 3.3 ZooMS reveals *Epinephelus aeneus* as the dominant species in northeastern Mediterranean grouper catches

In total, 84 (89%) of the 94 grouper samples were able to be identified to species level (Figure 3). All four Mediterranean grouper species within the *Epinephelus* genus were identified, with *Epinephelus aeneus* dominating the ancient grouper fishery. Using osteometrics, catch sizes were estimated for 17 of the fish with sizes ranging from 30 to 150 cm TL.

**FIGURE 4:**
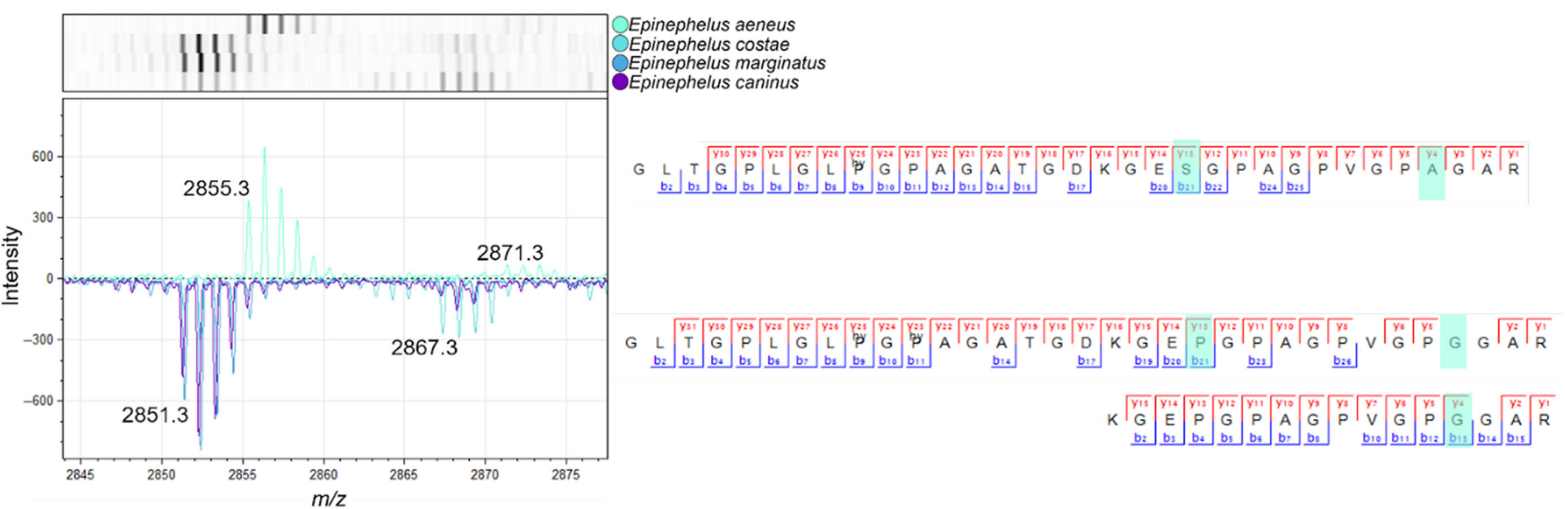
MALDI‐TOF peaks and amino acid sequence as recovered from LC–MS/MS of COL1ɑ1 586. *Epinephelus aeneus* has a sequence for the COL1ɑ1 586 peptide that is distinctive from the other Mediterranean groupers in this study.

The most informative peptide for identifications is COL1ɑ1 586 (Figure 4), ‘GLTGPLGLPGPAGATGDKGE**S**GPAGPVGP**A**GAR’ with a mass of *m/z* 2855/2871 and only present in *Epinephelus aeneus*. Another form of this peptide ‘GLTGPLGLPGPAGATGDKGE**P**GPAGPVGP**G**GAR’ with a mass of *m/z* 2851/2867 is present in the other three Mediterranean groupers in the *Epinephelus* genus (Figure 4). All of the samples identified as *Epinephelus aeneus* had the species‐specific form of the COL1ɑ1 586 peptide.

## 4 DISCUSSION

### 4.1 COL1 utility for Mediterranean grouper phylogeny and ZooMS identifications

Through the reconstruction of COL1 sequences, we have demonstrated that there is adequate variation in COL1 sequences to produce a well supported phylogenetic tree. Previously proposed phylogenies for eastern Atlantic groupers, including all four of our study species, were inconsistent (Maggio et al., 2005). The phylogeny we have proposed matches well with other genetic work done on our study species and the species included in our reference library (Vella et al., 2022). Our tree currently provides the best supported phylogenetic tree for *Epinephelus aeneus* and *Epinephelus caninus* in relation to other Mediterranean groupers. Due to the challenges and inaccuracies of phylogenies based on morphology, osteology and/or larval development, molecular approaches are necessary for elucidating grouper phylogenetic relationships (Ma & Craig, 2018).

We demonstrate that ZooMS can successfully be utilised for intra‐genus species‐level identification of Mediterranean groupers, demonstrating the research potential for high precision taxonomic resolution of archaeological fish remains using ZooMS. The seven indeterminate samples may represent poor preservation, the remaining Mediterranean grouper species, noting the regular presence of *M. rubra* in Iskenderun Bay today (Mavruk et al., 2018), or combers.

With the exception of COL1ɑ2 568 (1319.6 *m/z*), none of our novel ZooMS markers overlap with previously published (Harvey et al., 2022) ZooMS markers for Caribbean groupers (*Epinephelus* and *Mycteroperca*) or combers (Serranidae). Harvey et al. (2022) reports COL1ɑ2 568 (1319.6 *m/z*) being present in *Serranus tigrinus* and we find the same marker only in *E. caninus*. Therefore, in the absence of reference data for Mediterranean combers, we cannot exclude that this marker may be present in additional Serranidae species. This marker has thus not been relied on exclusively to identify archaeological samples. The reliability of the intra‐genus biomarkers we have found is supported by the quantity of distinctive biomarkers validated, the genetic distance between combers and groupers (Vella et al., 2022) and variability provided by the three distinctive collagen chains in Actinopterygii fish (Kimura & Ohno, 1987).

As demonstrated by one of our original reference assemblage samples which turned out to be a seabass (*Dicentrarchus* sp.) instead of a grouper, morphological identifications can be challenging and ensuring that reference samples have been accurately identified is essential. Furthermore, samples which have not been macerated or curated with enzymatic treatments (e.g. neutrase) are preferable for quality, reliable MALDI‐TOF spectra. Samples which have been treated with additional enzymes will not cleave in the same predictable manner as those which have only been processed with trypsin, resulting in unreliable MALDI‐TOF spectra. However, should samples of particular species be difficult to obtain (e.g. CITES permits, availability), such treated samples are still informative for LC–MS/MS analysis.

Applying these biomarkers to additional Mediterranean archaeological grouper remains could generate critical insights into how species distributions and abundances have changed over time. Species‐specific peptide biomarkers for Mediterranean groupers have the potential to aid in identifying species in present‐day fish markets (Dierickx et al., 2023; Mazzeo et al., 2008). As grouper meat is highly organoleptic, it is not uncommon for grouper in Mediterranean fish markets to be falsely labelled and substituted for cheaper fishes (Asensio et al., 2008; Cutarelli et al., 2014; Giusti et al., 2022; Trotta et al., 2005). The rate of mislabelling is likely to increase as additional Lessepsian species enter into the Mediterranean and indigenous species' geographical ranges change in the face of warming sea temperatures (Rilov, 2016).

### 4.2 *Epinephelus aeneus* dominance for millennia and revised growth capacity

The domination of *Epinephelus aeneus* in our archaeological assemblage suggests local *Epinephelus aeneus* population persistence since and throughout the Middle to Late Holocene within Iskenderun Bay. Our finding of long‐term *Epinephelus aeneus* abundance in this area is consistent with the current thought that this species is predominantly found in catches from along the Atlantic coast of Africa and southern and eastern Mediterranean coasts (Froese & Pauly, 2023) and has only recently been expanding their range northwards (Bañón et al., 2020). In present‐day Iskenderun Bay and the nearby Gulf of Mersin, *Epinephelus aeneus* is the most dominant species and forms a key resource for small scale fisheries (Mavruk, 2020; Mavruk et al., 2018, 2020; Özyurt & Kiyağa, 2016).

Ancient art and observations within well‐designed and enforced MPAs suggests that the natural habitat of large *Epinephelus marginatus* also includes shallow waters (<10 m; Guidetti & Micheli, 2011; Sala et al., 2012). It is suspected that large groupers may have a behavioural response of moving to deeper waters to become less targetable when fishing pressures are high (Guidetti & Micheli, 2011; Winter et al., 2022). Currently, large *Epinephelus aeneus* are only being found at great depths in the eastern Mediterranean (Özbek et al., 2013). The local *Epinephelus aeneus* resiliency we have detected may therefore be the result of this species shifting their habitat to deeper waters in the face of increasingly heavy fishing pressures. Further studies in MPAs in the eastern Mediterranean may provide additional insight into the ‘natural’ habitual depths of *Epinephelus aeneus*.

By combining our species‐level ZooMS identifications with reconstructed catch sizes, we are able to revise existing knowledge regarding the maximum body size of *Epinephelus aeneus*. Since *Epinephelus aeneus*'s recognition as a species in 1817 (Geoffroy Saint‐Hilaire, 1817), the reported maximum size is 120 cm TL (Froese & Pauly, 2023). Four of our archaeological samples from Kinet Höyük identified as *Epinephelus aeneus* had estimated catch sizes larger than this, ranging from 130 to 150 cm TL. By combining ZooMS identifications with osteometry, we have evidence that *Epinephelus aeneus* is capable of growing notably (ca. 30 cm) larger than previously recorded. This newly discovered amendment to *Epinephelus aeneus* growth capacity suggests potential for the maximum body size for other Mediterranean species may be revised with additional archaeological evidence. The modern absence of such large *Epinephelus aeneus* throughout their global distribution is the likely consequence of overfishing and ‘fishing down the food web’ (Pauly et al., 1998) to the point that no records or collective memories (Mavruk et al., 2018) exist of this species reaching such impressive sizes.

### 4.3 Contribution to the ongoing conservation efforts of Mediterranean groupers

Long established and well‐enforced MPAs have been shown to be successful for recovering abundance and sizes of groupers, resulting in increased fish biomass within MPA boundaries (Claudet et al., 2008; Guidetti et al., 2014; Hackradt et al., 2014). However, a key element to the ecological effectiveness of Mediterranean MPAs supporting high biodiversity and the recovery of overexploited taxa is the connectivity that occurs in regions where MPAs are in higher abundance and concentration, such as the northwestern Mediterranean Sea (Andrello et al., 2013). Restrictions on fisheries include limits on gear type, catch quotas, catch size limits and seasonal or spatial fishery closures. Fishery management, however, is difficult to enforce and presents additional challenges such as when banned species become bycatch (Mavruk et al., 2020; Silvano et al., 2017; Thiao et al., 2012). Such restrictions are also often met with resistance from small‐scale fisheries, as demonstrated by the 2016 ban prohibiting *Epinephelus aeneus* fishing in Turkey which was later repealed in 2018 (Mavruk, 2020). Reductions in fishing effort consequent to fisheries management plans and well‐managed MPAs can improve fishery outcomes, including increases in fishery yields and profits, which benefit local communities (Di Lorenzo et al., 2016; Hackradt et al., 2014; Weigel et al., 2014). The effective enforcement of MPAs and/or of other fishery management measures in Iskenderun Bay, in conjunction with ongoing efforts to restore the local habitat (Gökçe, 2015), are two crucial steps in ensuring the ongoing longevity and resilience of *Epinephelus aeneus* in Iskenderun Bay while also supporting local, sustainable grouper fisheries.

## 5 CONCLUSION AND FUTURE DIRECTIONS

Due to the paucity of reference data available, we have reconstructed four new Mediterranean grouper COL1 sequences, validated intra‐species variation with a phylogenetic tree created from COL1 sequences, and then identified 22 ZooMS biomarkers that can resolve grouper identification to the species level. Our ZooMS markers were able to identify 89% of specimens within the archaeological assemblage from Kinet Höyük. While our ecological baselines go back 5000 years, fishing in the Mediterranean extends at least 10,000 years back. By using these methods, we can ascertain the past biogeography, fisheries targets and catch sizes of additional vulnerable species and provide ecological baselines with greater geographical and temporal depth. Considering the available evidence, we cautiously hypothesise that the local *Epinephelus aeneus* population may have persisted for millennia. However, additional research focusing on the genetic connectivity of Mediterranean *Epinephelus aeneus* populations would be necessary to validate this hypothesis. Unexpectedly, we found ancient *Epinephelus aeneus* up to 30 cm larger than observed today, showing that we have not yet restored our Mediterranean marine ecosystems to match historical baselines, even in the most long‐established and effective MPAs in the Mediterranean Sea. Our study demonstrates the potential of ZooMS within ichthyoarchaeology and presents a framework for proteomic analyses to reconstruct the ecohistory of key marine taxa.

## AUTHOR CONTRIBUTIONS

**Rachel M. Winter:** Conceptualization (equal); data curation (lead); formal analysis (lead); investigation (lead); methodology (equal); project administration (lead); validation (lead); visualization (lead); writing – original draft (lead); writing – review and editing (lead). **Willemien de Kock:** Formal analysis (supporting); writing – review and editing (supporting). **Meaghan Mackie:** Data curation (equal); formal analysis (supporting); investigation (equal); writing – original draft (supporting); writing – review and editing (supporting). **Max Ramsøe:** Investigation (supporting); project administration (supporting); writing – original draft (supporting); writing – review and editing (supporting). **Elena Desiderà:** Formal analysis (supporting); visualization (supporting); writing – original draft (supporting); writing – review and editing (equal). **Matthew Collins:** Resources (equal); visualization (supporting); writing – review and editing (supporting). **Paolo Guidetti:** Formal analysis (supporting); supervision (supporting); writing – original draft (supporting); writing – review and editing (supporting). **Samantha Presslee:** Data curation (supporting); investigation (equal); resources (supporting); writing – review and editing (supporting). **Marta Munoz Alegre:** Data curation (supporting); investigation (supporting). **Tarek Oueslati:** Resources (equal); writing – review and editing (supporting). **Arturo Morales Muniz:** Resources (equal); writing – review and editing (supporting). **Dimitris Michailidis:** Resources (equal); writing – review and editing (supporting). **Youri van den Hurk:** Resources (supporting); writing – review and editing (supporting). **Alberto J. Taurozzi:** Conceptualization (equal); data curation (supporting); formal analysis (equal); investigation (equal); methodology (lead); resources (equal); supervision (equal); writing – original draft (equal); writing – review and editing (equal). **Canan Çakirlar:** Conceptualization (lead); formal analysis (supporting); funding acquisition (lead); project administration (equal); resources (lead); supervision (equal); writing – original draft (equal); writing – review and editing (equal).

## CONFLICT OF INTEREST STATEMENT

None of the authors have any competing interests to declare.

## Supporting information


Data S1:

## Data Availability

The mass spectrometry proteomics data have been deposited to the ProteomeXchange Consortium via the PRIDE partner repository (Perez‐Riverol et al., 2019) with the dataset identifier PXD042430. The protein sequence data reported in this paper appear in the UniProt Knowledgebase under the accession number(s): C0HM84, C0HM85, C0HM86, C0HM87, C0HM88, C0HM89, C0HM90, C0HM91, C0HM92, C0HM93, C0HM94 and C0HM95 and are additionally included in Data S1.
