# Supplementary material for: Grouping groupers in the Mediterranean: Ecological baselines revealed by ancient proteins
Source: Ecol Evol. 2023 Oct 23;13(10):e10625. doi: 10.1002/ece3.10625 (PMC10591212; doi:10.1002/ece3.10625)
Supplement: Supplementary file 1 — Data S1: [file ECE3-13-e10625-s001.docx]

# Supplemental Materials and Methods

## Protein extraction from modern and archaeological fish bones

Archaeological and modern grouper bones were sampled, using ca. 10 mg, in a keratin-exclusion laboratory at the Globe Institute of the University of Copenhagen. Protein extraction was with minor modifications from the method described in [(Buckley *et al.* 2009)](https://paperpile.com/c/NYq6Na/Yp3Vv). Samples were placed in 1.2M HCl at 4°C on a rotating rack until demineralized. Proteins were then extracted using a GuHCl extraction buffer, quantified with a BCA assay, and digested with Trypsin. As a final step before MALDI-TOF and LC-MS/MS analysis, extracted proteins (now in the form of peptides) were desalted by ZIPtipping.

## LC-MS/MS data acquisition

Leftover ZooMS Eluates were delivered to the Novo Nordisk Center for Protein Research, University of Copenhagen. 5-10 μL of eluate was transferred to a 96-well MS plate, based on concentration determined in the BCA step of the ZooMS extraction or from NanoDrop (Thermo Fisher Scientific) results of the eluate at 205 nm. To remove the ACN and concentrate the samples, samples were vacuum-centrifuged until approximately 3 μL of sample remained. Samples were then resuspended in 10 μL of 5% ACN 0.1% TFA.

Samples were then analysed by liquid chromatography-tandem mass spectrometry (LC-MS/MS) with an EASY-nLC 1200 (Proxeon, Odense, Denmark) connected to an Exploris 480 Orbitrap mass spectrometer (Thermo Scientific, Bremen, Germany). Sample separation was completed on a 15 cm column (75 μm inner diameter) in-house laser pulled and packed with 1.9 μm C18 beads (Dr. Maisch, Germany). 1-2 μL was injected for all samples based on sample concentrations. An integrated column oven was used to maintain the column temperature at 40°C. Buffer A was milliQ water and the peptides were separated with increasing buffer B (80% ACN and 0.1% FA) with a 77 min gradient, increasing from 5% to 30% in 50 min, 30% to 45% in 10 min, 45% to 80% in 2 min, held at 80% for 5 min and decreased back to 5% in 5 min and held for 5 min. Flow rate was 250 nL/min.

The Exploris 480 was operated in data-dependent top 10 mode. Spray voltage was set to 2kV, the heated capillary at 275°C, and the S-lens RF level was at 40%. Full scan mass spectra (MS1) were recorded at a resolution of 120,000 over the m/z range 350-1400 with a target AGC of 300% and a maximum injection time of 25 ms. HCD-generated product ions (MS2) were recorded with a maximum ion injection time set to 118 ms and a target ACG value of 200%. HCD collision energy was set at 30% and the isolation window was 1.2 m/z with the dynamic exclusion set to 20 s. A wash blank using 5% ACN 0.1% TFA was run in between each sample to hinder cross-contamination.

## Reconstruction of COL1 amino acid sequences

After initial reconstruction of Mediterranean *Epinephelus* spp. COL1 sequences, additional sequences were added to the reference library to improve coverage. Sequences of each reconstructed chain were run through BLASTp on NCBI and the top matches for each chain were added to the reference library. The additional sequences include: >XP_042361230.1, XP_049416991.1, XP_049925274.1, XP_053190734.1, XP_049457373.1, XP_049890956.1, XP_049418603.1, XP_049915244.1, XP_042363352.1.

## Full details of phylogenetic tree reconstruction

  The evolutionary history was inferred by using the Maximum Likelihood method and General Reversible Mitochondrial + Freq. model [(Adachi & Hasegawa 1996)](https://paperpile.com/c/NYq6Na/IPiou). The bootstrap consensus tree inferred from 10,000 replicates [(Felsenstein 1985)](https://paperpile.com/c/NYq6Na/xwwOO) is taken to represent the evolutionary history of the taxa analysed. Branches corresponding to partitions reproduced in less than 50% bootstrap replicates are collapsed. The percentage of replicate trees in which the associated taxa clustered together in the bootstrap test 10,000 replicates are shown below the branches [(Felsenstein 1985)](https://paperpile.com/c/NYq6Na/xwwOO). Initial tree(s) for the heuristic search were obtained automatically by applying Neighbor-Join and BioNJ algorithms to a matrix of pairwise distances estimated using the JTT model, and then selecting the topology with superior log likelihood value. A discrete Gamma distribution was used to model evolutionary rate differences among sites (5 categories (+G, parameter = 0.3415)). This analysis involved 8 amino acid sequences. All positions with less than 25% site coverage were eliminated, i.e., fewer than 75% alignment gaps, missing data, and ambiguous bases were allowed at any position (partial deletion option). There were a total of 4251 positions in the final dataset. Evolutionary analysis were performed in MEGA11 [(Tamura *et al.* 2021)](https://paperpile.com/c/NYq6Na/R1Kpt).

## Table S1. Archaeological samples for ZooMS analyses

Table S1. These fish bones come from hand collection and wet sieving through 2 mm mesh size. Catch size is reported as Total Length unless designated as Standard Length (SL) and was estimated following the methods outlined in (Winter *et al*. 2022).

| **Sample ID** | **Taxa based on osteomorphology** | **Catch Size*** | **Time Period** | **ZooMS identification** |
| --- | --- | --- | --- | --- |
| KT 10163 | Epinephelidae/Serranidae |  | MIA | *Epinephelus aeneus* |
| KT 11502 | Epinephelidae/Serranidae |  | MBA | *Epinephelus aeneus* |
| KT 12196 | Epinephelidae | >44.6 SL | LIA | *Epinephelus aeneus* |
| KT 12196 | Epinephelidae/Serranidae |  | LIA | *Epinephelus aeneus* |
| KT 14531 | Epinephelidae | >44.6 SL | LIA | *Epinephelus aeneus* |
| KT 14544 | Epinephelidae/Serranidae |  | LIA | *Epinephelus aeneus* |
| KT 16374 | Epinephelidae/Serranidae |  | MBA | *Epinephelus aeneus* |
| KT 20698 | Epinephelidae/Serranidae |  | EIA | *Epinephelus aeneus* |
| KT 20722 | Epinephelidae | >44.6 SL | EIA | *Epinephelus aeneus* |
| KT 20722 | Epinephelidae/Serranidae |  | EIA | *Epinephelus aeneus* |
| KT 20771 | Epinephelidae | >44.6 SL | EBIII | *Epinephelus aeneus* |
| KT 20771.1 | Epinephelidae/Serranidae |  | EBIII | *Epinephelus aeneus* |
| KT 21658 | Epinephelidae | 148.13 | MIA | *Epinephelus aeneus* |
| KT 21658.1 | Epinephelidae/Serranidae |  | MIA | *Epinephelus aeneus* |
| KT 23222 | Epinephelidae | 83.85 | LBII | *Epinephelus aeneus* |
| KT 23359 | Epinephelidae | >44.6 SL | LBII | *Epinephelus aeneus* |
| KT 23362 | Epinephelidae/Serranidae |  | LBII | *Epinephelus aeneus* |
| KT 23368 | Epinephelidae/Serranidae |  | LBA | *Epinephelus aeneus* |
| KT 23372 | Epinephelidae/Serranidae |  | LBA | *Epinephelus aeneus* |
| KT 23455 | Epinephelidae | 74.06 | LBII | *Epinephelus aeneus* |
| KT 23455 | Epinephelidae | 86.61 | LBII | *Epinephelus aeneus* |
| KT 23470.1 | Epinephelidae/Serranidae |  | LBII | *Epinephelus aeneus* |
| KT 23470.2 | Epinephelidae/Serranidae |  | LBII | *Epinephelus aeneus* |
| KT 23570 | Epinephelidae/Serranidae |  | LBII | *Epinephelus aeneus* |
| KT 23578.1 | Epinephelidae | 136.5 | LBA | *Epinephelus aeneus* |
| KT 23578.2 | Epinephelidae | 149.93 | LBA | *Epinephelus aeneus* |
| KT 23598 | Epinephelidae/Serranidae |  | LBA | *Epinephelus aeneus* |
| KT 23642 | Epinephelidae/Serranidae |  | LBA | *Epinephelus aeneus* |
| KT 23769 | Epinephelidae/Serranidae |  | LBA | *Epinephelus aeneus* |
| KT 23780.2 | Epinephelidae | 50.8 | LBII | *Epinephelus aeneus* |
| KT 23995 | Epinephelidae/Serranidae |  | LBA | *Epinephelus aeneus* |
| KT 24023 | Epinephelidae/Serranidae |  | LBII | *Epinephelus aeneus* |
| KT 24030.2 | Epinephelidae/Serranidae |  | LBII | *Epinephelus aeneus* |
| KT 24036 | Epinephelidae/Serranidae | >44.6 SL | LBA | *Epinephelus aeneus* |
| KT 24901 | Epinephelidae/Serranidae | >44.6 SL | LBA | *Epinephelus aeneus* |
| KT 24977 | Epinephelidae/Serranidae | >44.6 SL | LBA | *Epinephelus aeneus* |
| KT 3930 | Epinephelidae/Serranidae | about 44.6 SL | LBA | *Epinephelus aeneus* |
| KT 3930 | Epinephelidae/Serranidae |  | LBA | *Epinephelus aeneus* |
| KT 6239 | Epinephelidae/Serranidae |  | MED | *Epinephelus aeneus* |
| KT 6239.2 | Epinephelidae/Serranidae |  | MED | *Epinephelus aeneus* |
| KT 6369 | Epinephelidae | 94.65 | LIA | *Epinephelus aeneus* |
| KT 7053.1 | Epinephelidae | 132.55 | EIA | *Epinephelus aeneus* |
| KT 7113 | Epinephelidae/Serranidae |  | EIA | *Epinephelus aeneus* |
| KT 7339 | Epinephelidae/Serranidae |  | LIA | *Epinephelus aeneus* |
| KT 7344 | Epinephelidae |  | HELL | *Epinephelus aeneus* |
| KT 7641 | Epinephelidae | 88.39 | MIA | *Epinephelus aeneus* |
| KT 7641.2 | Epinephelidae | 84.58 | MIA | *Epinephelus aeneus* |
| KT 7727 | Epinephelidae/Serranidae |  | LIA | *Epinephelus aeneus* |
| KT 7841 | Epinephelidae | 94.44 | LIA | *Epinephelus aeneus* |
| KT 7841.1 | Epinephelidae/Serranidae |  | LIA | *Epinephelus aeneus* |
| KT 8074 | Epinephelidae |  | LIA | *Epinephelus aeneus* |
| KT 8446 | Epinephelidae/Serranidae |  | LBA | *Epinephelus aeneus* |
| KT 8482 | Epinephelidae | 72.24 | LIA | *Epinephelus aeneus* |
| KT 8487 | Epinephelidae | 62.73 | LIA | *Epinephelus aeneus* |
| KT 8595 | Epinephelidae/Serranidae |  | ? | *Epinephelus aeneus* |
| KT 8994 | Epinephelidae | 66.95 | LBII | *Epinephelus aeneus* |
| KT 8994.2 | Epinephelidae | >44.6 SL | LBII | *Epinephelus aeneus* |
| KT 8994.3 | Epinephelidae | >44.6 SL | LBII | *Epinephelus aeneus* |
| KT 9066 | Epinephelidae | 52.74 | LBII | *Epinephelus aeneus* |
| KT 9106.1 | Epinephelidae | >44.6 SL | LIA | *Epinephelus aeneus* |
| KT 9106.1 | Epinephelidae/Serranidae |  | LIA | *Epinephelus aeneus* |
| KT 9556 | Epinephelidae/Serranidae |  | MED | *Epinephelus aeneus* |
| KT 9747 | Epinephelidae/Serranidae |  | LBA | *Epinephelus aeneus* |
| KT 9794 | Epinephelidae |  | LBA | *Epinephelus aeneus* |
| KT 14430 | Epinephelidae/Serranidae |  | LIA | *Epinephelus caninus* |
| KT 21330.2 | Epinephelidae/Serranidae |  | EIA | *Epinephelus caninus* |
| KT 5955 | Epinephelidae/Serranidae |  | LIA | *Epinephelus caninus* |
| KT 8634 | Epinephelidae/Serranidae |  | LBA | *Epinephelus caninus* |
| KT 10893 | Epinephelidae/Serranidae |  | LIA | *Epinephelus caninus or E. costae* |
| KT 12343.2 | Epinephelidae/Serranidae |  | MBA | *Epinephelus caninus or E. costae* |
| KT 24030.1 | Epinephelidae/Serranidae |  | LBII | *Epinephelus caninus or E. costae* |
| KT 10344 | Epinephelidae/Serranidae |  | MIA | *Epinephelus marginatus* |
| KT 10858 | Epinephelidae/Serranidae | 30.15 | LBII | *Epinephelus marginatus* |
| KT 11134 | Epinephelidae/Serranidae |  | MIA | *Epinephelus marginatus* |
| KT 11237 | Epinephelidae/Serranidae |  | MIA | *Epinephelus marginatus* |
| KT 11564 | Epinephelidae/Serranidae |  | MBA | *Epinephelus marginatus* |
| KT 11569 | Epinephelidae/Serranidae |  | MBA | *Epinephelus marginatus* |
| KT 19420 | Epinephelidae/Serranidae |  | EBIII | *Epinephelus marginatus* |
| KT 23564 | Epinephelidae/Serranidae | <44.6 SL | LBA | *Epinephelus marginatus* |
| KT 23648.1 | Epinephelidae/Serranidae |  | LBII | *Epinephelus marginatus* |
| KT 23755 | Epinephelidae/Serranidae |  | LBA | *Epinephelus marginatus* |
| KT 23795 | Epinephelidae/Serranidae |  | LBII | *Epinephelus marginatus* |
| KT 23859 | Epinephelidae/Serranidae |  | LBII | *Epinephelus marginatus* |
| KT 4110 | Epinephelidae/Serranidae |  | LBA | *Epinephelus marginatus* |
| KT 6964 | Epinephelidae/Serranidae |  | LIA | *Epinephelus marginatus* |
| KT 9932 | Epinephelidae/Serranidae |  | LBII | *Epinephelus marginatus* |
| KT 10125 | Epinephelidae/Serranidae |  | MED | *Epinephelus costae* |
| KT 14896 | Epinephelidae/Serranidae | 35.41 | Late Hellenistic | indeterminate |
| KT 6376 | Epinephelidae/Serranidae |  | EIA | indeterminate |
| KT 8171 | Epinephelidae | >44.6 SL | EIA | indeterminate |
| KT 12847 | Epinephelidae/Serranidae |  | MED | indeterminate |
| KT 23365.2 | Epinephelidae/Serranidae |  | LBII | indeterminate |
| KT 24014 | Epinephelidae/Serranidae |  | LBII | indeterminate |
| KT 8357 | Epinephelidae/Serranidae |  | LBA | indeterminate |

## Table S2. Modern samples for LC-MS/MS and ZooMS analyses

The spectra obtained from samples which were macerated with papain did not appear abnormal whereas the sample prepared with neutrase did display a poor signal to noise ratio. Reference samples which have not been macerated or curated with enzymatic treatments (e.g., neutrase) are preferable for quality, reliable MALDI-TOF spectra. This is because samples which have been treated with additional enzymes will not cleave in the same predictable manner as those which have only been processed with trypsin (as is used for ZooMS), resulting in unreliable MALDI-TOF spectra. However, should samples of particular species be difficult to obtain (e.g., CITES permits and regulations, availability), treated samples are still informative for LC-MS/MS analysis.

When analysing archaeological grouper bones, there are smaller fishes, combers (Serranidae), with similar ecology that overlap in size with sexually immature groupers and are osteomorphologically indistinguishable from smaller groupers [(Desse & Desse-Berset 1996)](https://paperpile.com/c/NYq6Na/72OHD)). Combers were not included in our study due to belonging to a different taxonomic family to groupers [(Ma & Craig 2018; Vella *et al.* 2022)](https://paperpile.com/c/NYq6Na/eAGv4+HIjvr), making it unlikely for novel grouper biomarkers to also be present in combers.

| **Sample ID** | **Species** | **Origin** | **Lab** | **Collaborator** | **Maceration history and comments** |
| --- | --- | --- | --- | --- | --- |
| WLAP1086 | *Dicentrachus* sp.* | Athens, Greece | Malcolm H. Wiener Laboratory for Archaeological Science | Dimitris Michailidis | First identified using Whitehead et al. 1986, later (summer 2021) morphological analysis concluded the specimen belongs to the *Dicentrachus* genus. Specimen treated with acetone after macerating. |
| WLAp 1082 | *E. aeneus* | Athens, Greece | Malcolm H. Wiener Laboratory for Archaeological Science | Dimitris Michailidis | acetone suspected to have been used in specimen prepration. |
| AEN-1 | *E. aeneus* | Madrid, Spain | Laboratorio de Arqueozoologia at the Departamento de Biologia of the Universidad Autónoma de Madrid | Arturo Morales-Muñiz | macerated with boiling water. |
| AEN-3 | *E. aeneus* | Madrid, Spain | Laboratorio de Arqueozoologia at the Departamento de Biologia of the Universidad Autónoma de Madrid | Arturo Morales-Muñiz | macerated with boiling water. |
| CAN-1 | *E. caninus* | Madrid, Spain | Laboratorio de Arqueozoologia at the Departamento de Biologia of the Universidad Autónoma de Madrid | Arturo Morales-Muñiz | macerated with boiling water. |
| id765 | *E. caninus* | Tunisia | Département Histoire de L’Art et Archaeologie at Université de Lille | Tarek Oueslati | macerated with papaïne. |
| id1003 | *E. costae* | Greece | Département Histoire de L’Art et Archaeologie at Université de Lille | Tarek Oueslati | small specimen from spear fishing; macerated with water/ |
| ALE-1 | *E. costae* | Madrid, Spain | Laboratorio de Arqueozoologia at the Departamento de Biologia of the Universidad Autónoma de Madrid | Arturo Morales-Muñiz | macerated with boiling water. |
| id711 | *E. marginatus* | Tunisia | Département Histoire de L’Art et Archaeologie at Université de Lille | Tarek Oueslati | macerated with papaïne. |
| id449 | *E. marginatus* | Tunisia | Département Histoire de L’Art et Archaeologie at Université de Lille | Tarek Oueslati | macerated with papaïne. |
| GUA-4 | *E. marginatus* | Madrid, Spain | Laboratorio de Arqueozoologia at the Departamento de Biologia of the Universidad Autónoma de Madrid | Arturo Morales-Muñiz | macerated with boiling water. |
| ALE-4 | *E. costae* | Madrid, Spain | Laboratorio de Arqueozoologia at the Departamento de Biologia of the Universidad Autónoma de Madrid | Arturo Morales-Muñiz | neutrase used during maceration, MALDI-TOF data therefore disregarded. |
| GUA-7 | *E. marginatus* | Madrid, Spain | Laboratorio de Arqueozoologia at the Departamento de Biologia of the Universidad Autónoma de Madrid | Arturo Morales-Muñiz | macerated with boiling water. |
| GUA-8 | *E. marginatus* | Madrid, Spain | Laboratorio de Arqueozoologia at the Departamento de Biologia of the Universidad Autónoma de Madrid | Arturo Morales-Muñiz | macerated with boiling water. |

*This sample was initially included as one of the reference specimens as it had been identified as a white grouper (*E. aeneus*) when added to the Malcolm H. Wiener Laboratory for Archaeological Science’s reference collection in the 1990’s. Upon initial assessment of the MALDI-TOF spectra of all reference samples, this particular sample had numerous peaks which differed from all of the other grouper reference specimens. Subsequent osteological analysis of the WLAp 1086 skeleton at the Malcolm H. Wiener Laboratory for Archaeological Science revealed that this fish is actually a sea bass (*Dicentrarchus* sp.), not a grouper.

## Table S3 of ZooMS peaks for Mediterranean *Epinephelus* spp.

| **Mass (m/z)** | **Peptide Sequence** | **Peptide name** | ***E. aeneus*** | ***E. costae*** | ***E. marginatus*** | ***E. caninus*** |
| --- | --- | --- | --- | --- | --- | --- |
| 1319.6 | GE**G**GHRGPDGNAGR | COL1ɑ2 568 |  |  |  | **x** |
| 1350.6 | GE**A**GHRGPDGNAGR | COL1ɑ2 568 | x | x | x | x* |
| 1755.78 | GFTGMQGLPGPAG**A**HGER | COL1ɑ3 934 | x |  | x | x |
| 1783.8/1799.8 | GFTGMQGLPGPAG**V**HGER | COL1ɑ3 934 |  | **x** |  |  |
| 1935.9 | GLTGPIGLPGPAGATGDKGE**P**G | COL1ɑ1 586 |  |  |  | **x** |
| 1965.9 | GEPGPAGVQGL**S**GPSGEEGKR | COL1ɑ3 271 | x* | x* | x | x* |
| 1991.9 | GEPGPAGVQGL**P**GPSGEEGKR | COL1ɑ3 271 | x | x |  | x |
| 2178.0 | GF**S**GLPGPAGE**P**GKPGPSGPGGER | COL1ɑ1 793 | x* |  | x |  |
| 2178.0 | GF**P**GLPGPAGE**A**GKPGPSGPGGER | COL1ɑ1 793 | x |  | x* |  |
| 2309.1 | GLPGSPGSSGPPGKEG**A**AGP**A**GQDGR | COL1ɑ2 361 | x** |  | x | x |
| 2351.1 | GLPGSPGSSGPPGKEG**P**AGP**S**GQDGR | COL1ɑ2 361 |  | **x** |  |  |
| 2542.97 | VGPPGPSGNPGPPGPAGG**T**GKEGPKGNR | COL1ɑ1 705 |  | x** | **x** |  |
| 2537.6 | VGPPGPSGNPGPPGPAGG**P**GKEGPKGNR | COL1ɑ1 705 | x** |  |  | x** |
| 2731.1 | GFTGMQGLPGPAG**A**HGERGPAGASGPAGPR | COL1ɑ3 934 | x |  | x | x |
| 2775.0 | GFTGMQGLPGPAG**V**HGERGPAGASGPAGPR | COL1ɑ3 934 |  | **x** |  |  |
| 2851.34/2867.34 | GLTGPIGLPGPAGATGDKGE**P**GPAGPVGP**G**GAR | COL1ɑ1 586 |  | x | x | x |
| 2855.33/2871.33 | GLTGPLGLPGPAGATGDKGE**S**GPAGPVGP**A**GAR | COL1ɑ1 586 | **x** |  |  |  |
| 2814.4 | GPAGAQGAVGAPGPKGN**S**GDPGASGPKGEPGAK | COL1ɑ3 238 |  | x** |  |  |
| 2889.2 | GPAGAQGAVGAPGPKGN**N**GDPGASGPKGEPGAK | COL1ɑ3 238 | x** |  | x | x |
| 2915.3 | GPPGPMGPPGLAGAPGEPGREGSPG**N**EGSAGR | COL1ɑ1 817 | x** |  |  |  |
| 2947.3 | GPPGPMGPPGLAGAPGEPGREGSPG**N**EGSAGR | COL1ɑ1 817 | x** | x | x* |  |
| 2867.3 | GPPGPMGPPGLAGAPGEPGREGSPG**N**EGSAGR | COL1ɑ1 817 |  | x | x* | x |
| 2888.3 | GPPGPMGPPGLAGAPGEPGREGSPG**S**EGSAGR | COL1ɑ1 817 |  |  | x** |  |

Peptide names follow nomenclature proposed by [(Brown *et al.* 2021)](https://paperpile.com/c/0QPhLG/Noi5p).

*indicates a peak that is present in the MALDI due to the existence of an isobaric peptide.

**denotes a peptide which is present in the LC-MS/MS data but which is unable to be visualized in the MALDI-TOF data.

## MALDI peaks and amino acid sequences of peptide markers

## COL1ɑ2 568


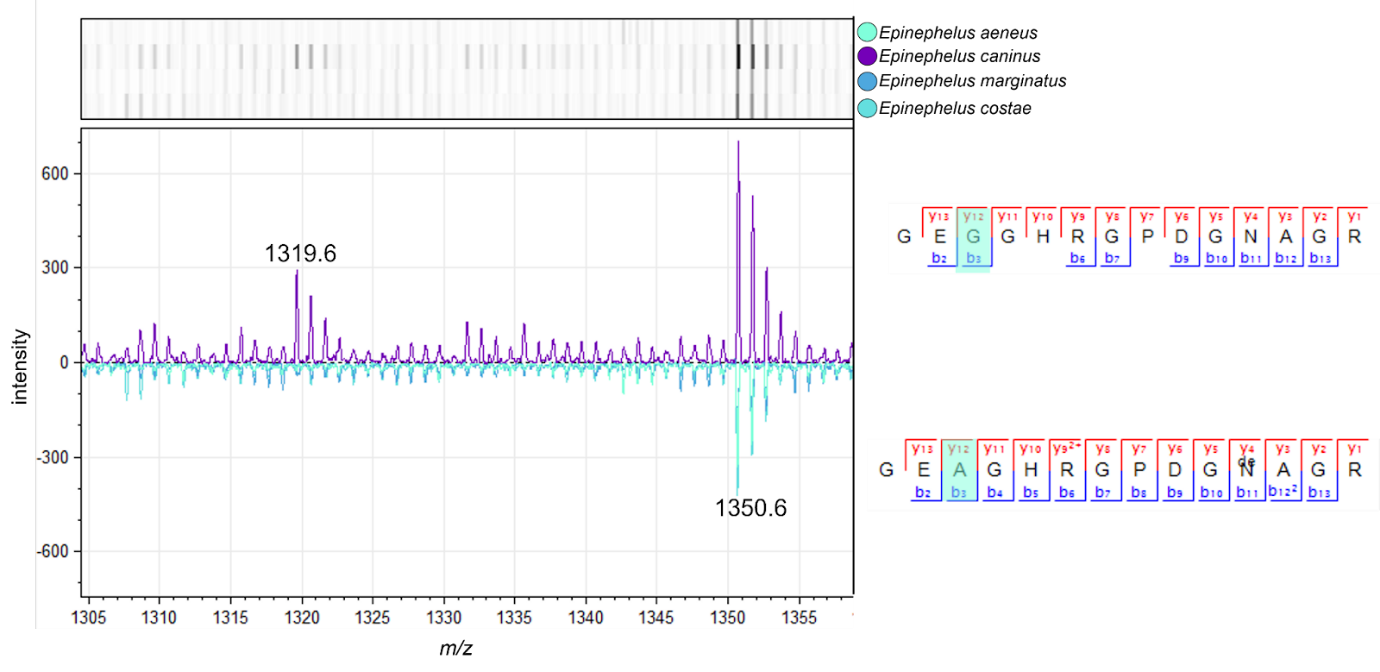


## COL1ɑ3 934 shorter peptide


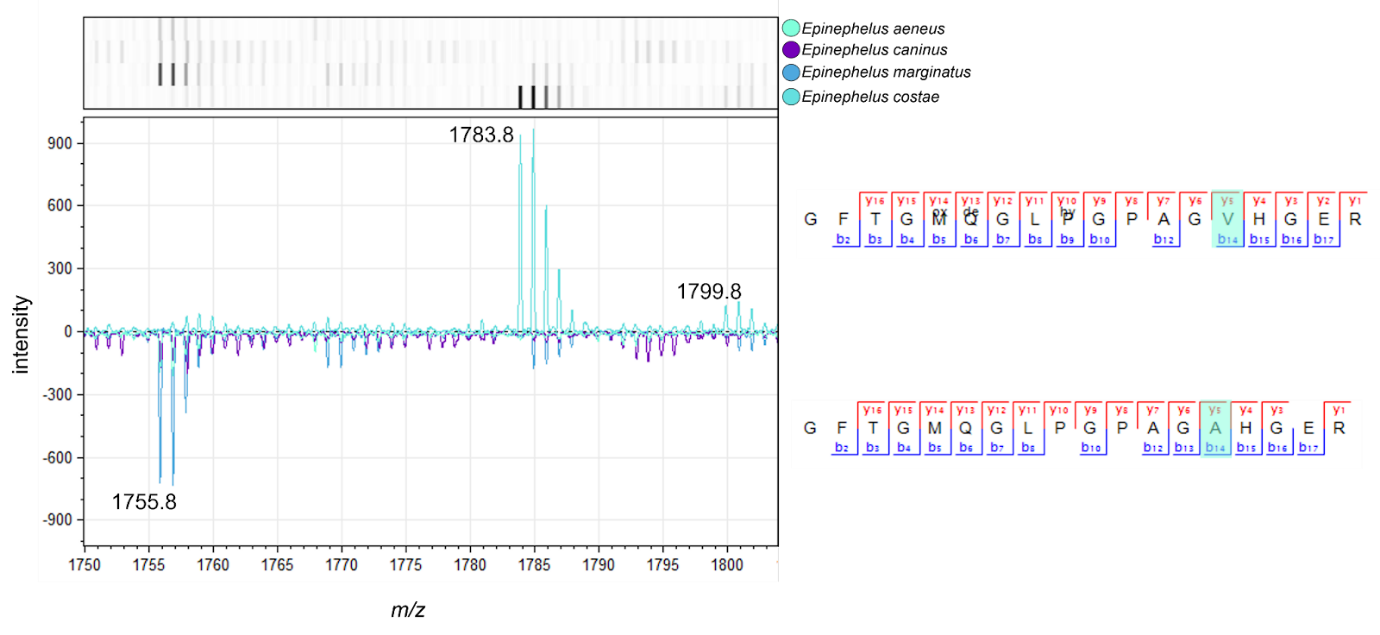


## COL1ɑ3 934 longer peptide


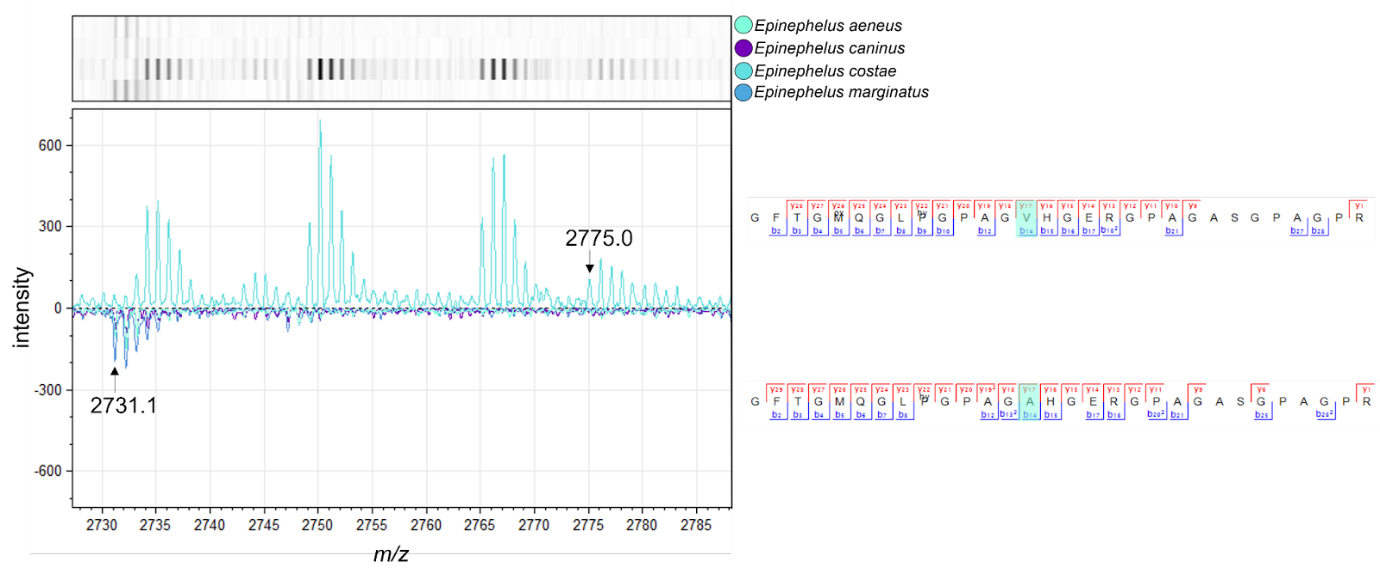


## COL1ɑ1 586


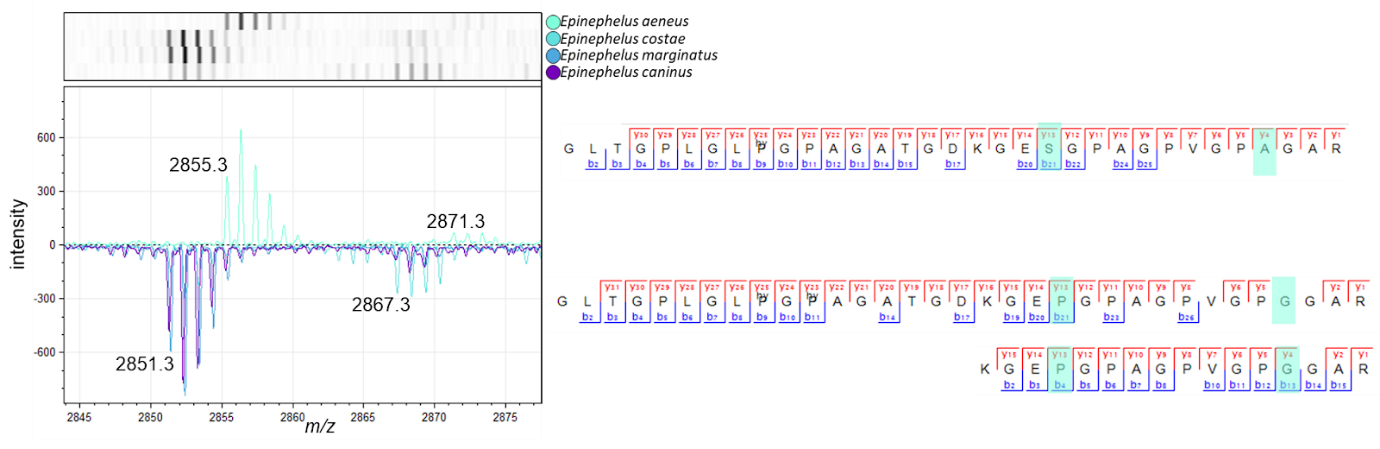


## COL1a3 271
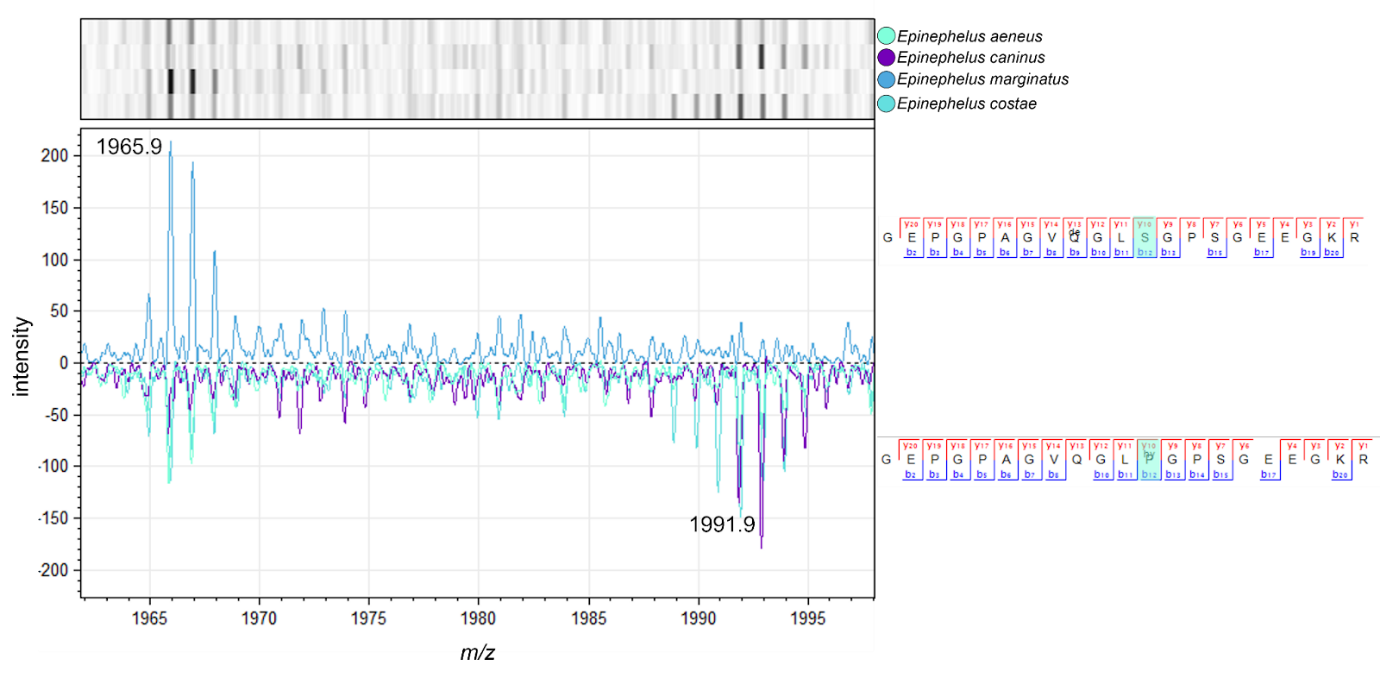


## COL1ɑ1 793


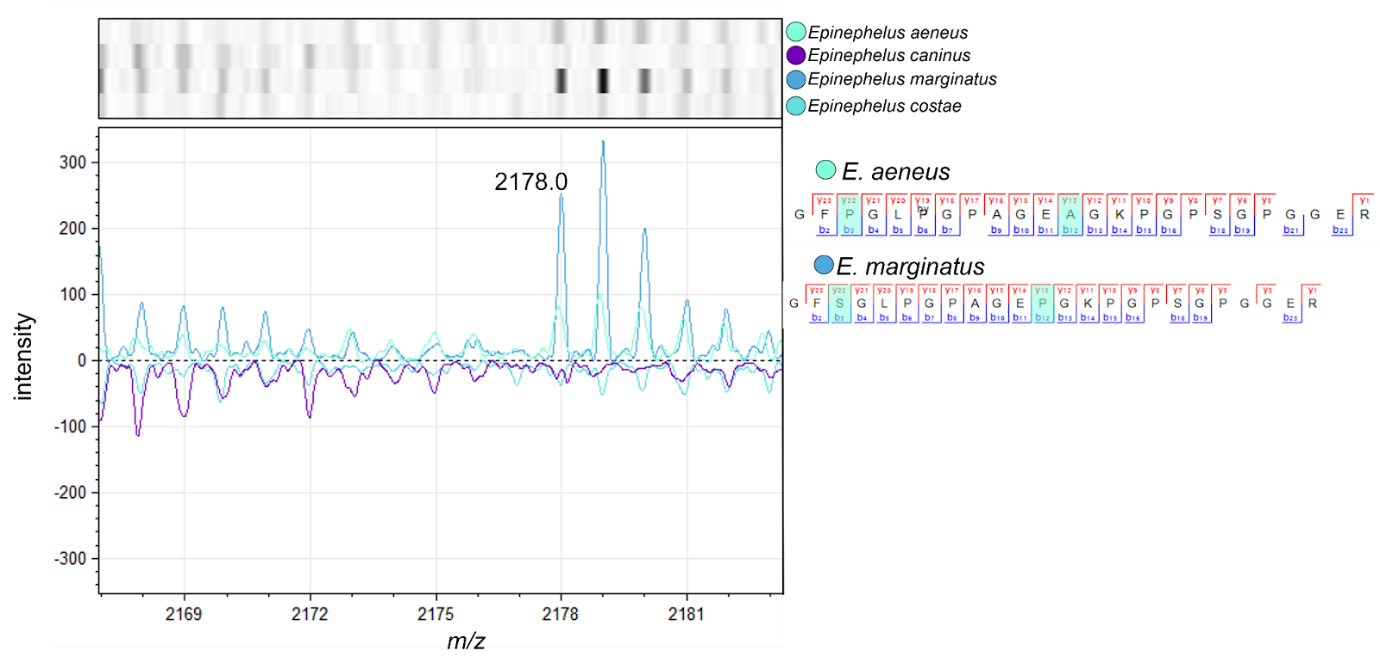


## COL1ɑ2 361


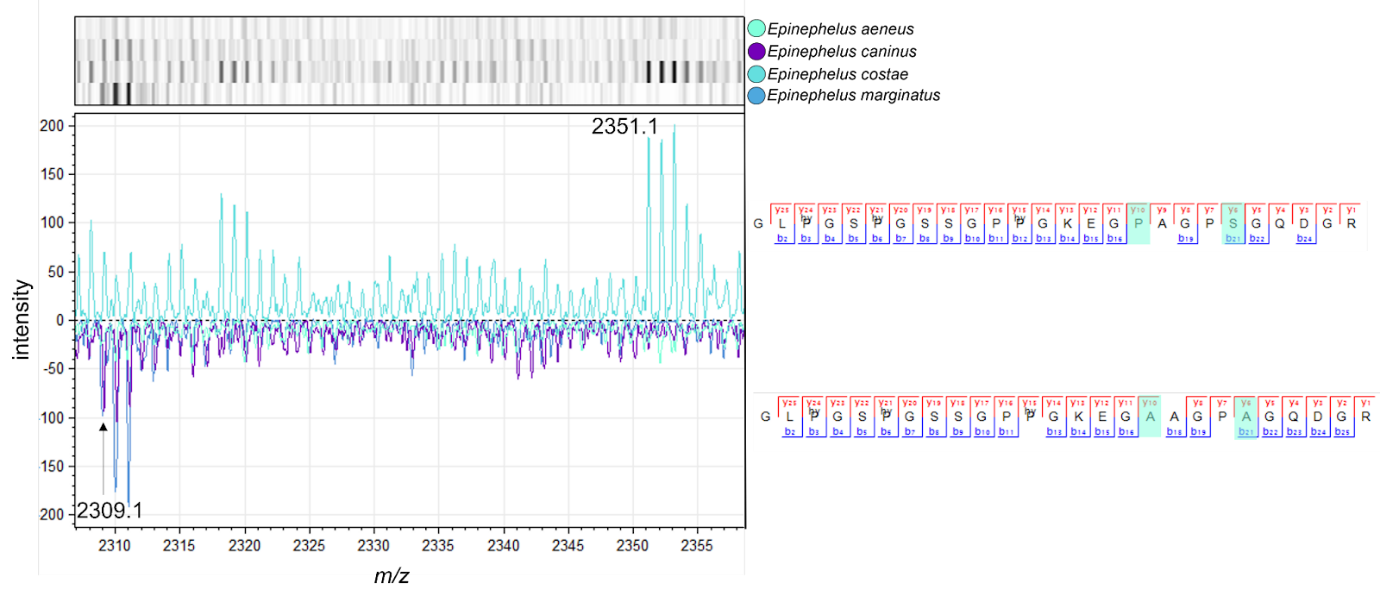


The ‘GLPGSPGSSGPPGKEG**A**AGP**A**GQDGR’ sequence in *E. caninus* and *E. marginatus* is also present in *E. aeneus* but the corresponding peptide mass is not present in the MALDI data for *E. aeneus*.

## COL1ɑ1 705


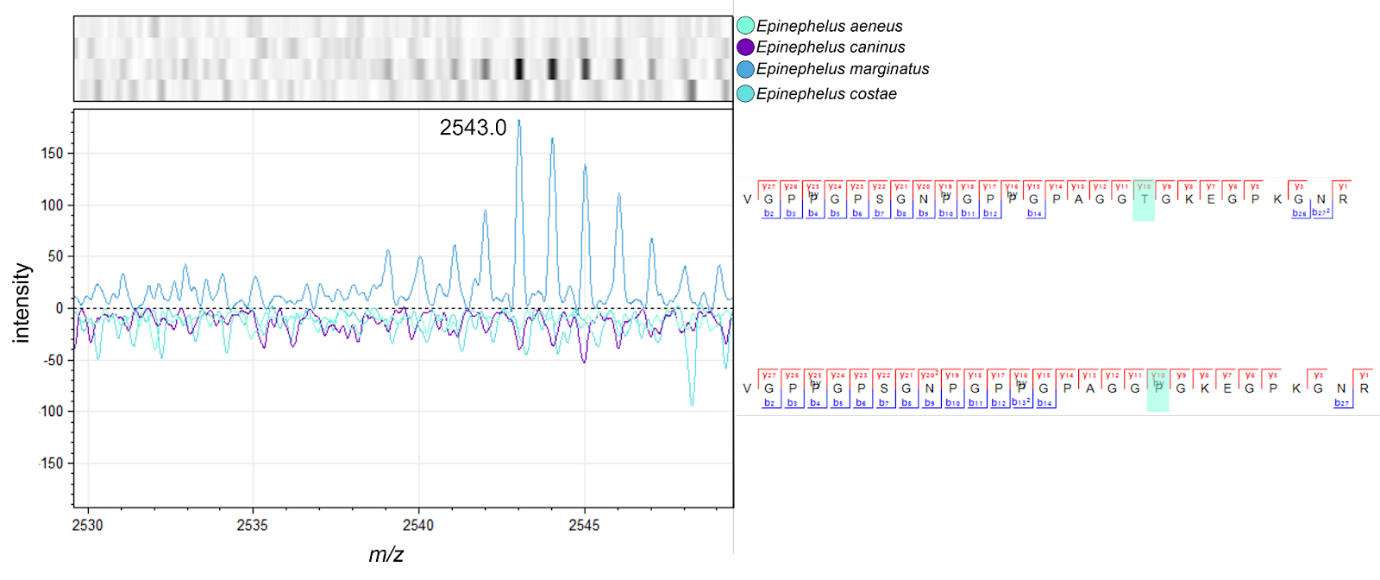


‘VGPPGPSGNPGPPGPAGG**P**GKEGPKGNR’ sequence is present in the LC-MS/MS data of *E. aeneus*, *E. caninus*, and *E. costae* but the corresponding peptide is not able to be visualized in the MALDI data.

## COL1ɑ3 238


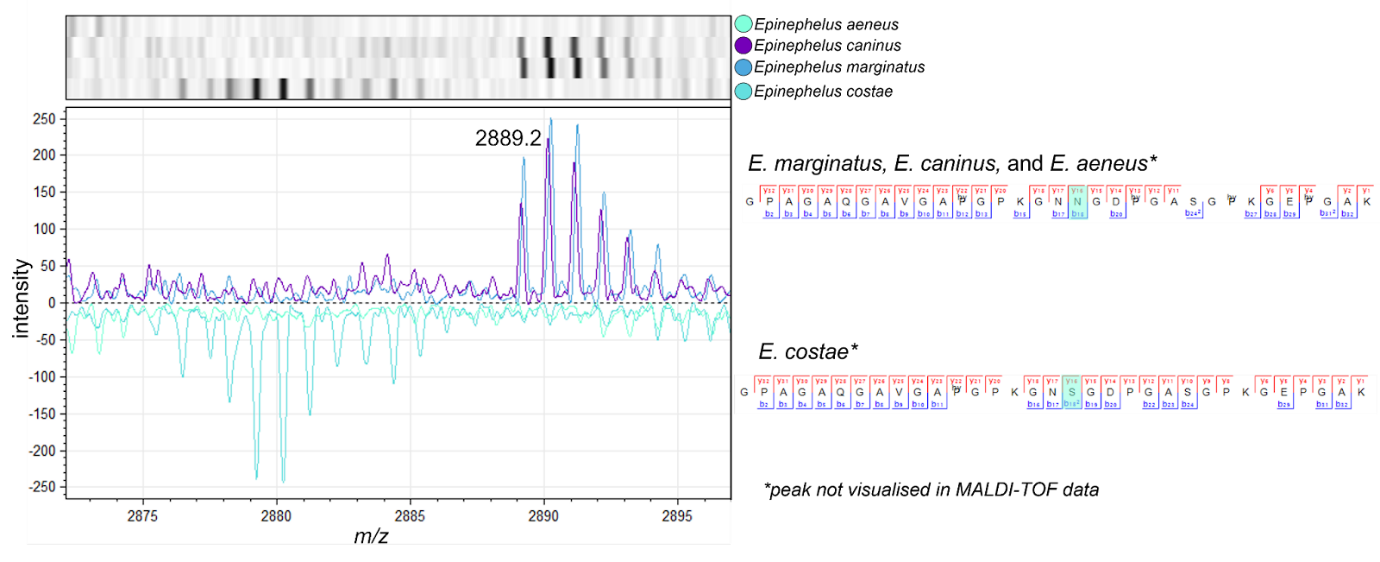


## References

[Adachi, J. & Hasegawa, M. (1996). Model of amino acid substitution in proteins encoded by mitochondrial DNA. *J. Mol. Evol.*, 42, 459–468.](http://paperpile.com/b/NYq6Na/IPiou)

[Brown, S., Douka, K., Collins, M.J. & Richter, K.K. (2021). On the standardization of ZooMS nomenclature. *J. Proteomics*, 235, 104041.](http://paperpile.com/b/NYq6Na/jZNGn)

[Buckley, M., Collins, M., Thomas-Oates, J. & Wilson, J.C. (2009). Species identification by analysis of bone collagen using matrix-assisted laser desorption/ionisation time-of-flight mass spectrometry. *Rapid Communications in Mass Spectrometry: An International Journal Devoted to the Rapid Dissemination of Up-to-the-Minute Research in Mass Spectrometry*, 23, 3843–3854.](http://paperpile.com/b/NYq6Na/Yp3Vv)

[Desse, J. & Desse-Berset, N. (1996). Archaeozoology of groupers (Epinephelinae). Identification, osteometry and keys to interpretation. *Archaeofauna*, 5, 121–127.](http://paperpile.com/b/NYq6Na/72OHD)

[Felsenstein, J. (1985). CONFIDENCE LIMITS ON PHYLOGENIES: AN APPROACH USING THE BOOTSTRAP. *Evolution*, 39, 783–791.](http://paperpile.com/b/NYq6Na/xwwOO)

[Ma, K.Y. & Craig, M.T. (2018). An inconvenient monophyly: an update on the taxonomy of the groupers (Epinephelidae). *Copeia*, 106, 443–456.](http://paperpile.com/b/NYq6Na/HIjvr)

[Tamura, K., Stecher, G. & Kumar, S. (2021). MEGA11: Molecular Evolutionary Genetics Analysis Version 11. *Mol. Biol. Evol.*, 38, 3022–3027.](http://paperpile.com/b/NYq6Na/R1Kpt)

[Vella, A., Vella, N. & Acosta-Díaz, C. (2022). The first complete mitochondrial genomes for Serranus papilionaceus and Serranus scriba, and their phylogenetic position within Serranidae. *Mol. Biol. Rep.*](http://paperpile.com/b/NYq6Na/eAGv4)
